# Models to quantify excretion of dry matter, nitrogen, phosphorus and carbon in growing pigs fed regional diets

**DOI:** 10.1186/2049-1891-4-42

**Published:** 2013-11-09

**Authors:** Henry Jørgensen, Trakarn Prapaspongsa, Van Thi Khanh Vu, Hanne Damgaard Poulsen

**Affiliations:** 1 Department of Animal Science, Aarhus University, Blichers Allé 20, P.O. Box 50, DK-8830 Tjele, Denmark; 2 Department of Civil and Environmental Engineering, Faculty of Engineering, Mahidol University, 25/25 Puttamonthon, Salaya, Nakorn Pathom 73170, Thailand; 3 National Institute of Animal Sciences, Thuy Phuong, Tu Liem, Hanoi, Vietnam

**Keywords:** Excretion, Faeces, Nitrogen, Phosphorus, Prediction, Urine

## Abstract

Modern pig production contributes to many environmental problems that relate to manure, especially in areas with highly intensive production systems and in regions like Asia where the regulative control is not effective. Therefore, the objective of this study was to use three different pig diets varying in dietary protein, fibre and fat as representative for Danish (DK), Thai (TH) and Vietnamese (VN) pig production to develop and evaluate different approaches to predict/calculate excretion from growing pigs in comparison with the experimentally determined values.

Nine female growing pigs were used in a digestibility and balance experiment. Excretion of dry matter (DM), nitrogen (N), phosphorus (P) and carbon (C) of the experimental diets were determined.

Due to the highest dietary fibre content, VN had the lowest digestibility of N, P and C (73, 49, and 73%, respectively) compared with the DK and TH pig diets. From the known diet composition using standard table values on chemical and nutrient digestibly, high accuracy (bias) and low variation was found and the results could be used for prediction on chemical composition and excretion in faeces and urine in growing pigs. Calculation based on standard values regarding nutrient retention in the pig body as used in the Danish manure normative system (DMNS) showed likewise to be quite useful for quantifying the total excretion of N and P.

Overall, the results demonstrate that simple models that require cheap and normally available information on dietary nutrients can give useful information on nutrient excretion in growing pigs.

## Background

Many environmental problems like surface water eutrophication, groundwater pollution, greenhouse gas emissions and odour relate to livestock manure especially in regions with intensive production systems. Highly intensive pig production has in many countries around the world resulted in a higher risk of negative environmental impact [1-3]. In fact, there are legislative measures to limit environmental impacts in many countries, e.g. Denmark, The Netherlands, and France where restrictions on animal density have been imposed [4]. However, the amount of nutrients in manure may exceed the amount that can be assimilated by crops resulting in nutrient accumulation in agricultural areas [4,5]. The situation is worse in Asian countries such as Thailand and Vietnam because there are no effective regulations or the regulations are poorly enforced [6].

Nevertheless, pig manure has potential for resource recoveries in terms of energy, such as biogas production, and nutrients, such as nitrogen (N) and phosphorus (P) fertilizers. The challenge is how to manage the resource recoveries more efficiently with lower effects on the environment. Many studies have shown that different feeding strategies can reduce nutrient excretions and greenhouse gas emissions by use of lowered dietary nutrient supplies adapted to the actual physiological requirements in pigs (i.e. phase feeding), use of synthetic amino acids to improve the utilization of crude protein, or microbial phytase supplementation combined with reductions in inorganic feed phosphates [7-10]. Nahm [9] reported that manure N can be decreased with up to 60% by the addition of synthetic amino acids to improve crude protein and the N utilization by 50% from 28 to 42% [11].

Tools to quantify inputs, outputs, and flows of nutrients at animal level is very useful for global design of manure management systems that efficiently take into account diet composition and productivity, resource recovery and environmental protection as well as economy. Therefore, the objective of this study was to use three different pig diets as representative for Danish (DK), Thai (TH) and Vietnamese (VN) pig production to develop different approaches to predict/calculate excretion from growing pigs in comparison with the experimentally determined values.

## Materials and methods

### *In vivo* experiments

Composite diets simulating practical diets used in Denmark (DK), Vietnam (VN) and Thailand (TH) with varied contents of dietary protein, dietary fibre and fat, respectively were formulated on the basis of feed ingredients purchased in Denmark (Table 1). Representative samples of diets were stored at -18°C for chemical analyses.

**Table 1:** Composition and chemical analysis of the experimental diets

**Diet**	**DK**	**VN**	**TH**
*Ingredients, %*			
Barley	26.04	-	-
Wheat	55.00	15.00	-
Oats	-	28.76	-
Pearl millet	-	-	42.50
Maize	-	-	22.05
Soybean meal	15.91	15.90	15.00
Wheat bran	-	15.00	10.00
Grass/Alfalfa meal	-	12.85	-
Vegetable oil	-	8.00	-
Fish meal	-	3.00	8.00
Limestone (CaCO_3_)	0.41	1.07	0.76
Salt	0.39	0.22	0.31
Dicalcium phosphate	1.00	-	1.18
Minerals and vitamins	0.20	0.20	0.20
Lysine, methionine, threonine mix	1.05	-	-

*DK* Danish diet, *VN* Vietnamese diet, *TH* Thai diet.

The experimental procedure was similar to Sørensen and Fernández [12]. Nine sibling female pigs were allocated individually to metabolism cages. Three growing pigs were subjected to two balance periods for each diet at 40 to 45 kg and 55 to 60 kg body weight - and fed 1.7 and 2 kg feed per d, respectively. Each balance period consisted of 5 d adaptation and 7 d complete collection of faeces and urine. Faeces and urine were collected quantitatively each d during the 7 experimental ds and stored at 5°C. Urine was collected through indwelling Foley catheters [13]. After 7 d the faeces collected from each pig were homogenized and samples were stored at -18°C until further analysis.

The diets, faeces and urine were analyzed chemically. Dry matter was determined by drying samples to a constant weight at 103°C, and ash was analyzed by incineration at 525°C. Nitrogen was measured by the Dumas procedure and protein was calculated as N × 6.25 [14]. Carbon was analyzed according to ISO-9831 [15]. P was determined by the vanadomolybdate colorimetric procedure [16]. Crude fat (HCl-fat) was extracted with diethyl ether after acid hydrolysis [17]. Crude fibre (CF) was assessed by the Weende method [18]. Starch was assayed by an enzymatic procedure according to Bach Knudsen [19] and sugar was analyzed by the method of Jacobsen [20].

### Calculations and statistical analyses

Based on the obtained chemical results, the digestibility, retention, utilization and excretions of N, P and C were determined according to Sørensen and Fernández [12] and shown in Table 2.

**Table 2:** Mean body weight, feed intake and experimentally determined nutrient balances and excretions in the in vivo experiment with pigs fed the three different diets (LS Mean values for six pigs)

**Composition**	**Diet**			**SE**^**1**^
	**DK**	**VN**	**TH**	
Mean body weight, kg	59.7	60.2	57.1	5.12
Feed intake, kg/d	1.80	1.78	1.43	0.21
Feed DM intake, kg/d	1.62	1.63	1.29	0.19
N				
N intake, g/d	45.5	51.2	49.2	4.23
N retention, g/d	21.2	23.5	21.2	2.03
Faecal N, g/d	8.79^a^	13.8^b^	10.0^ab^	1.33
Urine N, g/d	15.5	13.9	18.0	2.43
N excretion, % of intake	52.9	54.1	56.1	3.54
N Digestibility, %	80.6^a^	72.9^b^	79.9^a^	1.94
P				
P intake, g/d	9.17	8.55	9.99	0.84
P retention, g/d	3.91	4.12	4.34	0.38
Faecal P, g/d	4.39	4.37	4.41	0.42
Urine P, g/d	0.87^a^	0.05^b^	1.53^c^	0.22
P excretion, % of intake	57.2	51.8	59.0	2.69
P digestibility, %	51.9^ab^	48.7^a^	55.9^b^	2.21
C				
C intake, g/d	705^ab^	770^a^	573^b^	53.3
Faecal C, g/d	108^a^	208^b^	93^a^	12.5
Urine C, g/d	19.8	20.7	20.7	2.83
C excretion, % of intake	18.2^a^	29.7^b^	19.5^a^	1.00
C digestibility, %	84.6^a^	72.9^b^	84.1^a^	1.03

*DK* Danish diet, *VN* Vietnamese diet, *TH* Thai diet.

^1^Pooled standard error.

LS Means values within a row with the same letter are not significantly different at *P*<0.05 (Least Square Means test).

^abc^: Values in a row with different superscripts differ significantly (*P*<0.05).

The Danish manure normative system (DMNS) calculating N, P, and potassium (K) contents in manure has been established in order to provide Danish farmers and authorities with tools for fertilizer planning and control. The system calculates the nutrient flows by considering *ex animal*, *ex housing*, and *ex storage* contents of N, P and K [21]. First, the system includes standard values for dietary nutrient content, nutrient digestibility, feed intake, and nutrient retention in the pig body in order to calculate the excretion of the nutrients (*ex animal*). Then, the system accounts for losses due to emissions during housing to get *ex housing* values and finally, losses from emissions and denitrification during storage are subtracted (*ex storage*). In the present study, the excretion of N and P was calculated for a standard (mean) Danish pig based on the current mean values for dietary protein (N) and P content, digestibility of protein and P, daily feed intake, and daily N and P retention to give the actual daily excretion of N and P for a standard Danish growing-finishing pig in the interval from 30 to slaughtering at 105 kg [21-23].

Validation of the predicted/estimated results (Tables 3 and 4) was done by test set validation [24] using the present experiment. The performance of the prediction was evaluated by its prediction error in terms of root mean square error of prediction (RMSEP):

RMSEP=∑i=1nyi^-yi,ref2n

**Table 3:** Analyzed and calculated chemical composition and digestibility of nutrients of the experimental diets (DK, VN and TH)

	**Analyzed/Estimated ( *in vivo *)**			**Calculated from feedstuff tables**^**1**^				
	**DK**	**VN**	**TH**	**DK**	**VN**	**TH**	**Deviation**^**2**^	**RMSEP**^**3**^
*Chemical composition, % DM*								
Dry matter, %	90.1	91.5	90.0	86	89	87	3.2	0.5
Ash	5.0	5.9	5.7	5.7	6.2	6.6	-0.6	0.2
Crude Protein (N × 6.25)	17.5	19.6	23.8	17.5	19.3	22.6	0.5	0.4
Crude fat	3.1	13.3	5.6	2.4	12.5	4.0	1.0	0.3
Starch and sugar	56.2	32.6	48.2	54.7	35.8	48.5	-0.7	1.4
Total dietary fibre	18.3	28.6	16.7	16.9	27.5	15.0	1.4	0.2
Crude fibre	4.2	9.8	3.7	4.1	9.4	3.4	0.3	0.1
Phosphorus	0.57	0.52	0.77	0.64	0.55	0.77	-0.06	0.02
*Total digestibility, %*								
Dry Matter (DM)	85	73	85	82	71	82	2.3	0.4
Organic Matter (OM)	86	74	86	86	75	87	-0.9	0.4
Carbon	85	73	84	84^4^	72	85	0.1	0.6
Protein	81	73	80	83	76	82	-2.6	0.3

*DM* Dry matter, *DK* Danish diet, *VN* Vietnamese diet, *TH* Thai diet.

^1^Bach Knudsen [19], Just et al. [25], NRC [26] Vils and Sloth [27].

^2^Deviations, systematic difference between the calculated using table values and the experimental estimated value.

^3^RMSEP, root mean square error of prediction.

^4^Carbon digestibility calculated by the equation: dcCarbon (%) = -12.15 +1.117 dcOM (%), using data from Jørgensen [28].

**Table 4:** ***In vivo *** **estimation and calculated/predicted amount of excreted faeces DM, N, C and urine N of the experimental diets (DK, VN and TH)**

	***In vivo *** **estimation**				**Calculated from tables **^**1**^					**Predicted from equations**^**2**^				
	**DK**	**VN**	**TH**	**SE**^**3**^	**DK**	**VN**	**TH**	**Deviation**^**4**^	**RMSEP**^**5**^	**DK**	**VN**	**TH**	**Deviation**	**RMSEP**
Faeces DM, kg/d	0.24	0.44	0.20	0.027	0.28	0.46	0.22	-0.022	0.01	0.29	0.40	0.23	-0.012	0.03
Faeces N, g/d	8.8	13.8	10.0	1.33	7.4	11.7	8.1	1.82	0.2	9.3	12.5	9.2	0.52	0.6
Faeces C, g/d	108	208	93	12.5	105	209	85	3.4	3	114	170	82	14.6	13
Urine N, g/d	15.4	13.9	18.0	2.43	18.0^6^	18.7	18.5	-2.59	1.2	18.0	21.2	22.6	-4.81	1.4

*DK* Danish diet, *VN* Vietnamese diet, *TH* Thai diet.

^1^Based on feed intake shown in Table 2 and values calculated from tables (Bach Knudsen [19]; Just et al. [25]; NRC [26]; Vils and Sloth [27]) shown in Table 3.

^2^Based on feed intake shown in Table 2 and equations from Vu et al. [29].

^3^Pooled standard error.

^4^Deviation, systematic difference between the experimental estimated value and that calculated using either table values or predicted values.

^5^RMSEP, root mean square error of prediction.

^6^Urine N calculated assuming a 50% utilization of the digested N.

Bias represents the average difference between predicted and measured Y-values for all samples in the validation or reference data set (ref) and measure the accuracy of the prediction model. If there is no systematic difference between the average values of the two data sets, the deviation (bias) will be zero:

DeviationBias=∑i=1nyi^-yi,refn

## Results and discussion

### *In vivo* experiment with pigs fed Danish (DK), Vietnamese (VN) and Thai (TH) based diets

The chemical composition of the experimental diets is shown in Table 1. Generally, the protein and fat content were higher in the VN and TH pig diets compared with the DK diet whereas the fibre content was highest in the VN diet compared with the TH and the DK diets.

In general, no health problems were observed among the pigs throughout the experiment. Average feed intake for the DK and the VN was almost identical whereas the TH intake was lower (Table 2). Feed refusals were observed for the TH group during the first period and these pigs consumed 20% less than the other groups, which might be related to the inclusion of pearl millet that is known to contain tannins affecting palatability and reducing feed intake [30]. Therefore, vanilla flavour was added to TH during the second period resulting in increased feed intake to almost the same level as the VN and DK diets. The average body weight gain of pigs fed the TH diet was lower than the VN and DK pigs reflecting the lower feed intake of the pigs fed the TH diet without added flavour.

The analyzed contents for most nutrients reflected the calculated contents (Table 3) showing that table values on nutrient contents are quite reliable for the most common feedstuffs. Main feedstuffs in TH were maize and sorghum which are not typically used for pig feeding in Denmark and the use of these feedstuffs resulted in minor deviations from the standard values.

### Nutrient digestibility, retention and excretion

Nutrient balances in terms of intake, retention and excretion per d, total excretion in percentage of intake and the digestibility of dietary nutrients are summarized in Table 2. The digestibility of N, P and C in the TH and DK diets was very much alike whereas the digestibility was the lowest in the VN diet which might be related to the high crude fibre content in feed. The low P digestibility in VN might also be caused by the fact that feed phosphate (dicalcium phosphate, DCP) was solely added to the DK and TH diets, and it is known that DCP has a higher P digestibility than plant feedstuffs [31]. Previous studies have shown that high fibre levels decrease nutrient and energy digestibility in pigs [32-34] and increase fermentation and excretion of methane (CH_4_) to the environment [28]. Fibre can hinder the access of digestive enzymes to the cell contents [35] and can furthermore increase the passage rate of digesta [36]. This may also decrease the digestibility of nutrients and energy because of less access and time available for the digestive enzymes.

The N, P and C excretions in faeces and urine are presented in Table 2 and differed to some extend between diets although the total daily excretion of N and P did not differ significantly between diets. In contrast, the total C excretion was 50% higher in VN compared with DK and TH which was due to a much lower C digestibility in VN. The present study did not result in statistical differences in the urinary N and C excretion between the experimental diets whereas the faecal excretion of N and C was significantly different. The opposite was true for the P excretion. The retention of N and P was almost the same for all diets and was similar to the values of growing pigs reported by Fernández et al. [37] (21.0 g N and 4.15 g P per d). Excreted urinary P represents excessive dietary P in relation to the pigs’ physiological requirement. However, the small amount of excreted P in urine for VN can be regarded as obligatory losses (Table 2), but it seems likely that VN provided sufficient available P to fulfil the pigs’ P need. In general, excessive protein (N) and P intake results in higher daily excretion of N and P in urine. Many studies show that reductions in unavailable and/or excess N and P in diet can decrease the excretion of N and P [38-40].

### Calculations and predictions of excretions of faeces, N and C

In addition to the experimentally obtained results, two different models were used to quantify the excretions of faeces DM, N and C. First, the excretions were quantified using published table values on nutrient contents in the feedstuffs used in the *in vivo* experiment. Proximate analysis and digestibility of the used feedstuffs were derived from Just *et al.*[25], except pearl millet [26], cereals [27] and dietary fibre [19]. The calculated results are shown in Table 3.

Second, the excretions of faeces, DM, N and C were predicted using published equations. Vu et al. [29] proposed equations to calculate amounts of faeces and faeces composition derived from datasets of 285 diets assayed in digestibility experiments at the Department of Agricultural Sciences, Aarhus University. Vu et al. [29] showed that the calculated values using these equations did not differ significantly between equations with one, two or three parameters. Therefore, Vu et al. [29] defined the following criteria for parameterization of the equations, (i) easily obtainable parameters, (ii) as few parameters as possible, and (iii) a diminutive difference between the calculated and the experimental determined results. The selected equations from Vu et al. [29] used in the present study are shown below.

Faeces DM (kg/d) = -0.105 + 0.118 × DM intake (kg/d) + 0.00110 × DF (g/kg DM).

Faeces N (g/d) = 0.685 + 0.0260 × DF (g/kg DM) + 0.0855 × N intake (g/d).

Faeces C (g/d) = -98.82 + 68.95 × DM intake (kg/d) + 0.541 × DF (g/kg DM).

There were two sets of equations to calculate urinary N given by Vu et al. [29]. The equation representing dietary protein contents from 15 to 26% of DM and protein retention between 70 to 160 g/d was selected for the present study.

**Figure 1:**
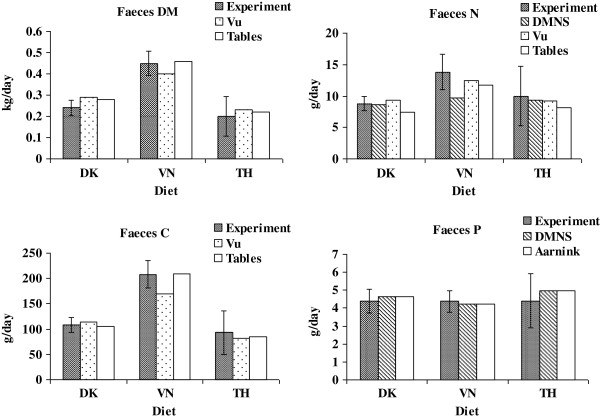
**Comparison of the daily excretion of faeces DM, N, C and P obtained in the present experiment (Experiment) with the Danish manure normative system (DMNS), Vu et al. [**[29]**] (Vu), calculated amounts based on table values (Tables) and Aarnink et al. [**[41]**] (Aarnink).** The experimental values are expressed as least square means (n = 6) with pooled standard errors. *DK* Danish diet, *VN* Vietnamese diet, *TH* Thai diet.

**Figure 2:**
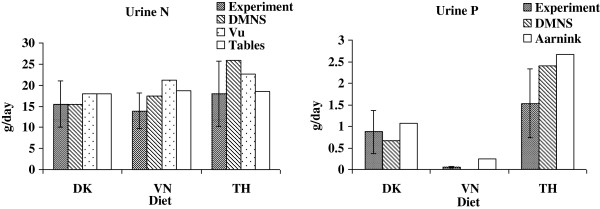
**Comparison of daily urine excretion of N and P obtained in the present experiment with the modified Danish manure normative system (DMNS), Vu et al. [**[29]**] (Vu), calculated amounts based on table values (Tables) and Aarnink et al. [**[41]**] (Aarnink).** The experimental values are expressed as least square means (n = 6) with pooled standard errors. *DK* Danish diet, *VN* Vietnamese diet, *TH* Thai diet.

**Figure 3:**
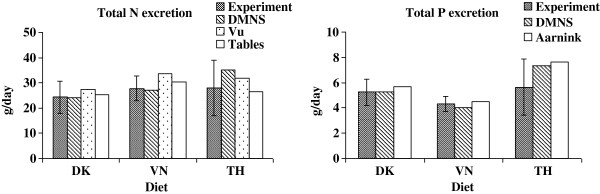
**Comparison of daily total N and P excretion obtained in the present experiment (Experiment) with the modified Danish manure normative system (DMNS), Vu et al. [**[29]**] (Vu), calculated amounts based on table values (Tables) and Aarnink et al. [**[41]**] (Aarnink).** The experimental values are expressed as least square means (n = 6) with pooled standard errors. *DK* Danish diet, *VN* Vietnamese diet, *TH* Thai diet.

Urine N (g/d) = -28.50 + 0.143 × Crude protein (g/kg DM) + 13.23 × DM intake (kg/d). The predicted values are shown in Table 4 and Figure 1, 2 and 3.

Comparing the analyzed values and values obtained from feedstuff tables on dietary nutrients is shown in Table 3. In general, the difference is very small which resulted in correspondingly small deviations when the digestibility of DM, OM, C and N (protein) was calculated based on either the analyzed values or standard values from feedstuff tables [19,25-27]. Thus, the cheap and quick approach using table values seems reasonable for obtaining indicative values on digestibility.

### Calculation of the daily excretions by use of table values or equations

The predicted amount of daily excretion of faeces DM, N, C and urine N using either information from tables [19,25-27] or equations [29] is shown in Table 4 and Figure 1 and 2. In general both methods of prediction show values within the standard error (SE) for the measured *in vivo* values. However, the variance (RMSEP) was smaller when using table values than when using equations. Bias or accuracy for prediction of faeces DM was negative for both methods showing a slight overestimation in average 7 and 3% when predicted were based on table values or equations, respectively. Faeces N were underestimated with 17% using the information from tables because of an underestimation of the N digestibility (Table 3). However, using the equations the bias was much smaller. Contrary to faecal N the prediction of faecal C showed an underestimation of 2% when using tables and 11% when prediction was based on equations by Vu et al. [29]. However, the predicted N excretion in urine based on Vu et al. [29] was higher (especially in the VN diet) than in the experiment with 17 and 30% of bias for tables and equations, respectively (Table 4 and Figure 2). The predictions assume an average utilization of 50% of the digested N which can be expected in average in practice [25,42]. In the current experiment, the utilization of N was higher (mean 58% N retained of digested N; Table 2) and as urinary N normally is higher than faecal N, it is evident that a reduction in N excretion can be obtained by feeding the pigs close to their requirement. Biases in total N excretion (1 to 17%) were mainly influenced by the N content in urine (Figure 3); however, the predictions were within the standard error found in the present experiment.

### Calculation of the daily excretions by use of the Danish manure normative system (DMNS)

The N and P balances in terms of intake, retention and excretion are shown in Table 5 and are compared with the experimental or calculated values in Figure 1, 2 and 3. Table 5 shows the actual mean values on N and P balances for a Danish grower-finisher pig (DK standard) in comparison with the calculated N and P balances based on the DMNS system for the experimental pigs fed DK, VN or TH in the present experiment. In general, the DK and VN diets mimicked the DK Standard, whereas the TH pigs showed a much higher excretion of N and P due to the higher feed conversion ratio (feed intake per kg gain).

**Table 5:** Calculated intake, retention and excretion of nitrogen (N) and phosphorus (P) based on the Danish Manure Normative System (DMNS) using standard values or the experimental values for DK, VN and TH treatments regarding N and P contents in the diets and feed intake

**Diet**	**DK standard**^**1**^	**DK**	**VN**	**TH**
Weight gain, g/d	840	719	810	465
Feed intake, kg/d	2.26	1.80	1.78	1.43
*N:*				
N intake, g/d	52.7	45.4	51.1	49.0
N retention, g/d	24.9	21.3	24.0	13.8
N excretion, g/d (total)	27.8	24.1	27.1	35.2
In faeces, g/d	10.0	8.63	9.70	9.31
In urine, g/d	17.8	15.5	17.4	25.9
*P:*				
P intake, g/d	9.18	9.24	8.47	9.91
P retention, g/d	4.62	3.96	4.45	2.56
P excretion, g/d (total)	4.56	5.29	4.02	7.35
In faeces, g/d	4.59	4.62	4.02	4.95
In urine, g/d	0.03	0.67	0^2^	2.40

^1^Mean values for the weight interval 30 to 105 kg. Diet content: protein 145.8 g/kg (23.3 g N/kg); phosphorus 4.06 g/kg. Digestibility: protein 81%; phosphorus 50%. Retention per kg gain: 29.6 g N; 5.5 g P (Fernández [22]).

^2^The urinary excretion was calculated to be-0.22 g/d and was set to 0.

In addition, the excretion of N and P was calculated for the experimental diets (DK, VN, TH) using the same principles but by use of the recorded daily intake and the dietary protein (N) and P content (given by the calculated composition from tables (Table 3)) in order to mimic the situation on a farm where the farmer has the declared dietary contents but does not know the actual retention and digestibility of the nutrients. The results from the estimation of N and P based on DMNS are shown in Table 5 and Figure 1, 2 and 3. Aarnink et al. [41] also estimated the P excretion in pig manure and proposed an equation for calculation of P retention that accounts for the effects of physiological stage (P retention = 0.005467 × W^-0.025^ × daily gain, g where W is the body weight of the pig). Otherwise, the Aarnink et al. [41] equations correspond to the DMNS equations. The excretion of P was also calculated by use of Aarnink et al. [41] and is shown in Figure 1, 2 and 3.

### Comparison of the experimental results with the model results

The experimental results on N, P, C and DM excretions are compiled and compared with predictions from (i) DMNS (regarding N and P), (ii) Vu et al. [29] (regarding DM, N and C), (iii) calculated amounts based on table values (regarding DM, N and C), and (iv) Aarnink et al. [41] (regarding P) in Figure 1, 2 and 3. Figure 1 shows that the predictions of faecal DM and P content for all models fall within the standard error seen in the experiment for all diets, but the DMNS model was not able to predict the faecal N excretion and the Vu et al. [29] equation was not able to predict the faecal C excretion within the experimental standard errors indicating that more variation of predicting faecal N and C can be expected. Thus, the different models seem to be quite valid for predictions of faecal DM and P excretions. Furthermore, the success of the models to predict faecal N and C excretion depended on the type of diet. Generally, the predictions of urinary excretions of N and P only showed results within the experimentally determined standard errors for DK and not for VN or TH (Figure 2). In contrast, all the models resulted in predictions of the total N and P excretions that fell within the experimentally obtained standard errors (Figure 3). Thus, all the tested models could be used to predict the N and P excretions in these diets representing regional different pig diets. Taken as a whole, the very simple models (DMNS and Aarnink et al. [41] which are principally very alike) were quite useful to predict the overall excretion of N and P whereas the equations given by Vu et al. [29] resulted in more precise predictions for the separate excretions of N and P in urine and faeces. Vu et al. [43] also shows that models based on the equations proposed by Vu et al. [29] are suitable for predictions of nutrient contents in manure for pigs fed Vietnamese diets. Although the present experiment was of limited duration and number of diets, it is anticipated that the conclusions can be expanded to a longer period reflecting e.g. the grower-finisher period of pigs.

This study also emphasizes that both the simple models and the more complex models may be used for evaluation of the potential for improvements in nutrient utilization and thus reductions in nutrient excretions. The provision of essential amino acids has been used to lower the protein contents in pig diets while maintaining adequate supply of essential amino acids without negative effects on pig performance [40,44]. However, this potential has not been fully utilized worldwide.

Thus, reducing protein contents in DK, VN and TH may be helpful in order to decrease the N excretion but this may not always be possible at a local or regional scale due to the supply of feedstuffs or economy. Similarly, substitution of feed phosphate by phytase may also lessen the P excretion, but this requires specific knowledge of the effects of phytase on P digestibility when microbial phytase is added to different diets composed of regionally relevant feedstuffs. Johansen and Poulsen [45] showed that the effects of microbial phytase highly depended on diet composition and the presence of plant phytase in the feedstuffs. Generally, the effect of phytase addition on P digestibility was greatest in feedstuffs with a low plant phytase activity. Nevertheless, the review showed a maximum P digestibility of not more than 60 to 65% when microbial phytase was supplemented to pig diets fed dry [45].

## Conclusion

This study showed that regional differences in diet composition simulated by three diets significantly affected manure characteristics. Due to the highest dietary fibre content, VN had the lowest digestibility of N, P and C (73, 49, and 73%, respectively) compared with the DK and TH pig diets. Very simple input–output models using either standard table values of the feedstuffs or standard values regarding nutrients retention in the pig body (like DMNS) seem quite useful in order to quantify the total excretion of N and P whereas the newly developed equations derived from datasets of almost 300 diets were very useful to predict the divided excretions of DM, N and C in faeces and in urine. In conclusion, these simple models seem to be quite robust and thus very useful as they are based on parameters and information that are available at a low cost under practical conditions. However, more experimental data have to be available and integrated if the effects of e.g. microbial phytase additions should be included in a further refined model.

## Abbreviations

DMNS: Danish manure normative system; RMSEP: Root men square error of prediction; DF: Total dietary fibre calculated as the residual fraction after subtraction of the analysed content of sugar, starch, crude protein, crude fat and ash from the dry matter.

## Competing interests

The authors declare that they have no competing interests.

## Authors’ contributions

TP and VTKV carried out the experimental trial, performed the statistics and drafted the manuscript. HJ and HDP participated in design and coordination of the study. All authors read and approved the final manuscript.
